# Pathophysiological Links Between Myocardial Infarction and Anxiety Disorder, Major Depressive Disorder, Bipolar Disorder and Schizophrenia

**DOI:** 10.3390/biology14040336

**Published:** 2025-03-25

**Authors:** Leong Tung Ong, Ching-Hui Sia

**Affiliations:** Department of Cardiology, National University Heart Centre, Singapore 119228, Singapore; ong.leong.tung@mohh.com.sg

**Keywords:** myocardial infarction, psychiatric disorders, anxiety disorder, major depressive disorder, bipolar disorder, schizophrenia, cardiovascular disease

## Abstract

There is increasing evidence demonstrating that psychiatric conditions elevate the risk of developing accelerated atherosclerosis and early-onset cardiovascular disease (CVD), including myocardial infarction (MI). Several mechanisms contribute to this observation. The dysfunction of the autonomic nervous system and hyperactivity of the hypothalamic–pituitary–adrenal axis in these patients contribute to the development of MI. Additionally, patients with underlying psychiatric disorders often have abnormal levels of anti-inflammatory and pro-inflammatory cytokines, which can lead to early vascular damage and subsequent atherosclerosis. Elevated PAI-1 levels, reduced tPA activity, and the decreased brain-derived neurotrophic factor (BDNF), influenced by coagulation and inflammation, may contribute to depression and its link to MI. Oxidative stress, marked by increased reactive species and impaired antioxidant defenses, is associated with cellular damage and has been consistently implicated in schizophrenia and bipolar disorder, potentially contributing to myocardial infarction. Finally, molecular genetic studies have indicated that psychiatric disorders and myocardial infarction may share potential pleiotropic genes. The interplay between the psychiatric conditions and myocardial infarction underscores the importance of integrated care approaches to manage both mental and physical health.

## Simple Summary

This review explores the association between myocardial infarction (MI) and major psychiatric conditions, such as depression, bipolar disorder, and schizophrenia. Research shows that people with psychiatric illnesses are at a higher risk of developing heart disease, and vice versa. This relationship is influenced by factors such as chronic inflammation, stress hormone imbalances, genetic predisposition, and oxidative stress, which can damage both the heart and brain. For example, stress and inflammation in MI can lead to changes in brain chemicals that can contribute to depression, while oxidative damage in psychiatric conditions can contribute to heart disease. Additionally, genetic factors may play a role in both conditions, further strengthening their association. Understanding these shared mechanisms is essential for improving the treatment of individuals with both MI and psychiatric disorders. This review highlights the need for further research and better medical strategies to reduce the impact of these conditions and improve the quality of life for affected individuals.

## 1. Introduction

Myocardial infarction (MI) and psychiatric conditions are two major causes of disability and death worldwide [1]. Many studies have demonstrated that the survivors of MI are at an elevated risk of developing psychiatric disorders and vice versa [1]. However, the exact neurobiological pathways that link MI to psychiatric illness remain poorly understood [2]. Individuals with major psychiatric conditions have a life expectancy that is approximately 15 to 25 years shorter than that of the general population, with cardiovascular disease accounting for the majority of these premature deaths [3,4]. Cardiovascular disease is the leading cause of death among individuals with major psychiatric conditions with mortality rates being more than twice compared to the general population, and this has continued to rise over recent decades [5].

The relationship between myocardial infarction and cardiovascular disease appears to be bidirectional, with acute coronary events and chronic cardiovascular conditions potentially triggering the development of psychiatric conditions [6]. In addition, the evidence indicates that even among patients with low coronary artery calcium scores, the mortality rates in those with major psychiatric conditions remain three to four times higher than in the general population [7]. The emerging research highlights the shared pathophysiological mechanisms between myocardial infarction and psychiatric conditions, encompassing biological, neurohormonal and genetic factors [8]. This review aims to explore the molecular mechanisms between myocardial infarction and major psychiatric conditions.

## 2. Anxiety Disorder and Major Depressive Disorder

**Figure 1:**
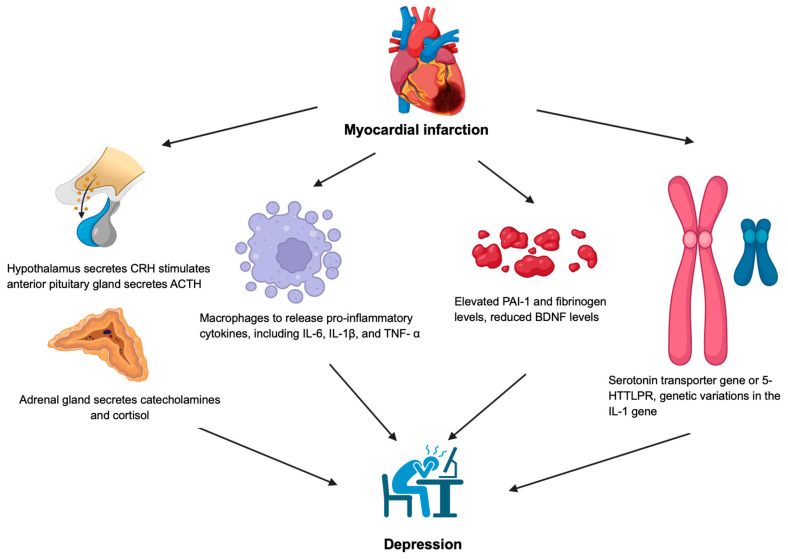
Pathophysiology of myocardial infarction and depression. ACTH: adrenocorticotropic hormone, BDNF: brain-derived neurotrophic factor, CRH: corticotropin-releasing hormone, IL: interleukin; PAI-1: plasminogen activator inhibitor 1, TNF-α: tumor necrosis factor-α; 5-HTTLPR: serotonin transporter gene variant.

Figure 1 demonstrates the pathophysiology of myocardial infarction and depression. Studies have demonstrated that patients with MI have a higher risk of developing newly diagnosed anxiety and major depressive disorders [9]. MI serves as a significant risk factor for newly diagnosed clinical anxiety and depressive disorders within the first two years following the event [9]. The biopsychosocial model suggests that the interplay of biological, psychological, and social factors may contribute to the development of anxiety and depressive disorder post-myocardial infarction [10]. MI can lead to major depressive disorder facilitated by increased symptoms of psychological discomfort post-MI cardiac rehabilitation compared to the general population [11].

MI triggers responses like the hypothalamic–pituitary–adrenal (HPA) axis activation and autonomic nervous system (ANS) dysregulation, potentially leading to prefrontal cortex and anterior cingulate gyrus dysfunction, which contributes to depression and anxiety disorder [12]. In addition, MI activates the secretion of the corticotropin-releasing hormone (CRH) from the hypothalamus, stimulating the anterior pituitary gland to release the adrenocorticotropic hormone (ACTH) [2]. The ACTH stimulates the adrenal glands to produce cortisol, catecholamines, and an abnormal cortisol level leading to HPA axis dysfunction due to impaired feedback control. This study demonstrated that the cortisol levels in post-MI patients spike immediately after the event due to the HPA axis activation but return to normal within 72 h [13]. While post-MI patients with depression lasting over three months showed a flattened daily cortisol rhythm, those without depression had significantly lower afternoon cortisol levels compared to the morning [13]. Abnormal cortisol rhythms have been associated with cognitive impairment and diminished stress-coping abilities, potentially heightening the risk of developing major depressive disorder and anxiety disorder [13,14].

Inflammation is one of the main pathophysiological mechanisms in the development of depression in patients post-MI [15]. During MI, damage-associated molecular patterns (DAMPs), like heat shock proteins (HSPs) and high-mobility group box 1 (HMGB1), are released from the heart muscles due to oxygen deprivation [15]. These molecules activate the immune cells, such as the macrophages that release pro-inflammatory cytokines, including interleukin (IL)-6, IL-1β, and the tumor necrosis factor (TNF)-α, which is essential for the phagocytosis of damaged cardiac muscle and cardiac repair [15,16]. Inflammatory cytokines are implicated in the development of depression, as demonstrated in both animal and human studies [12]. Research in rodent models reveals that inflammatory cytokines, such as IL-1β and TNF-α, can induce depressive-like behavior, which is also observed in humans where elevated inflammatory markers may lead to a depressed mood [17,18]. Inflammatory cytokines can disrupt neurotransmitter systems, such as reducing serotonin levels via increased indoleamine 2,3-dioxygenase activity, and altering dopamine and norepinephrine metabolism and production leading to the dysregulation of mood [19,20,21]. In addition, inflammatory cytokines activate the kynurenine pathway, which increases quinolinic acid production, which activates the N-methyl-D-aspartate (NMDA) receptor leading to the development of depressive symptoms [22].

The plasminogen activator inhibitor 1 (PAI-1), encoded by the SERPINE1 gene, serves as a key inhibitor of the tissue plasminogen activator (tPA) in the extracellular space, and has been associated with an increased risk of depression and the response of selective serotonin reuptake inhibitors [23,24]. PAI-1 levels increase during periods of psychological stress and depression, while serotonergic antidepressant treatment has been associated with reduced PAI-1 levels [23,25]. Additionally, patients with depression often exhibit lower baseline tPA levels, which significantly increase following antidepressant therapy, highlighting a potential relationship between depression and MI [26]. Elevated PAI-1 and fibrinogen levels may inhibit fibrinolysis leading to the evaluated risk of MI [12]. The coagulation system may lead to the development of depression through the tPA-plasmin pathway, which converts the pro-Brain-derived neurotrophic factor (BDNF) into BDNF, a neurotrophin essential for synaptic plasticity and neuronal connectivity [27]. In addition, increased inflammatory cytokines have also been shown to decrease levels of BDNF [28,29]. Reduced BDNF levels, particularly in the brain regions involved in mood regulation, like the amygdala, prefrontal cortex, hippocampus, and amygdala, have been consistently linked to emotional stress and depression [30].

Mendelian randomization studies have demonstrated a significant link between a genetic predisposition to depression and a higher risk of developing MI [12]. Otte et al. identified an association between the serotonin transporter gene variant (5-HTTLPR) and an increased risk of depression in patients with myocardial infarction (MI), with carriers of this variant exhibiting a poorer response to treatment with antidepressants [31]. In addition, patients with both depression and MI have an increased sensitivity or upregulation of serotonin receptors and a reduced expression of the serotonin-transporter receptor leading to increased thromboembolic risk [32]. Furthermore, the S allele of the 5-HTT gene polymorphic region has been linked to both depressive symptoms and adverse cardiac outcomes [33]. The genetic differences in the IL-1 gene were demonstrated to have a higher likelihood of developing depression following an MI, potentially due to the increase in the inflammatory response during MI [34].

## 3. Bipolar Disorder

**Figure 2:**
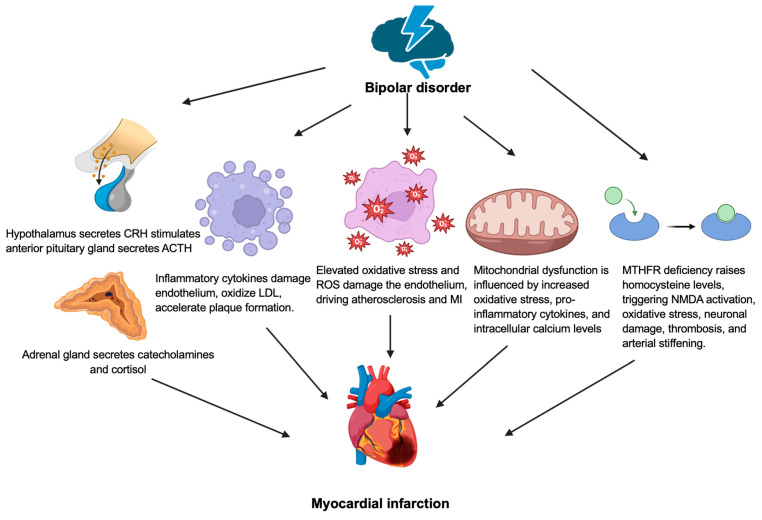
Pathophysiology of myocardial infarction and bipolar disorder. ACTH: adrenocorticotropic hormone, CRH: corticotropin-releasing hormone, LDL: low-density lipoprotein, MI: myocardial infarction, MTHFR: methylenetetrahydrofolate reductase, NMDA: N-methyl-D-aspartate, ROS: reactive oxygen species.

Figure 2 shows the pathophysiology of myocardial infarction and bipolar disorder. Patients with bipolar disorder experienced prolonged stress due acute mania or depression leading to a disrupted parasympathetic response [35]. The heart–brain axis allows communication between the cardiovascular and nervous systems, with sympathetic and parasympathetic activity mediated by acetylcholine, epinephrine, and norepinephrine to regulate cardiac contractility and heart rate variability [36,37]. Limited research has reported that patients with bipolar disorder have decreased heart rate variability, a key indicator of autonomic nervous system activity [38,39]. Reduced heart rate variability is associated with a 32–45% higher risk of the development cardiovascular disease [40]. During the acute phase of mania or depression in bipolar disorder, the HPA is activated, leading to the paraventricular nuclei to the secret CRH [35]. This stimulates the anterior pituitary gland to produce ACTH, leading to elevated cortisol levels [35]. Hypercortisolemia contributes to insulin resistance and hyperglycemia, leading to the increase in the release of pro-inflammatory cytokines [35]. The inflammatory cytokines damage the endothelial cells, accelerate the formation of the atherosclerotic plaques due to the oxidization of low-density lipoprotein (LDL), leading to cholesterol crystal build-up and MI [41].

The neurobiological basis of bipolar disorder may involve dysfunctions in the neurotrophic pathways and energy metabolism, with increased oxidative stress causing lipid and protein peroxidation, impairing signal transduction, structural plasticity, and cellular resilience [42]. In vivo magnetic resonance spectroscopy studies demonstrated altered levels of phosphocreatine, phosphomonoesters, and intracellular pH, highlighting the disruptions in oxidative phosphorylation, energy production, and phospholipid metabolism in bipolar disorders [42]. The acute phase of bipolar disorder leads to increased oxidative stress, characterized by elevated levels of oxidants, including peroxides and malondialdehyde, and reduced antioxidants, such as vitamin E, coenzyme Q10, and catalase that results in generating reactive oxygen species (ROS) through the pathways involving redox factor 1, activator protein 1, and the hypoxia-inducible factor 1 [43,44]. This oxidative damage contributes to endothelial wall injury, promoting atherosclerosis and cardiovascular diseases [45]. In addition, lipid peroxidation, driven by excess ROS damages polyunsaturated fatty acids in cell membranes, leading to the formation of lipid hydroperoxides and cellular dysfunction [46]. This oxidative damage can compromise mitochondrial integrity, disrupt energy metabolism, and trigger inflammatory responses, contributing to the development and progression of MI [46].

Mitochondrial dysfunction in bipolar disorder is influenced by increased oxidative stress, pro-inflammatory cytokines, and intracellular calcium levels, which are more pronounced during manic episodes [47]. Increased calcium ions and oxidative stress enhance oxidative phosphorylation through the pathways involving adenosine triphosphate (ATP) synthase, AMP-activated protein kinase (AMPK), SIRT-1, SIRT-3, and NAD+ [48]. Mitochondrial dysfunction in bipolar disorder, leading to excessive calcium and oxidative stress, may exacerbate myocardial injury in individuals with underlying ischemic heart disease. Excessive calcium entry into the mitochondria via the mitochondrial calcium uniporter (MCU) and impaired extrusion by the Na^+^/Ca^2^^+^ exchanger (NCX) results in mitochondrial calcium overload, leading to the opening of the mitochondrial permeability transition pore (MPTP) [49]. This, combined with oxidative stress-induced damage to mitochondrial membranes and proteins, results in ATP depletion, mitochondrial swelling, and cardiomyocyte death [50]. These processes exacerbate myocardial injury and infarct.

Bipolar disorder is associated with a deficiency of the methylenetetrahydrofolate reductase (MTHFR) enzyme, which is essential for homocysteine metabolism leading to increased central and peripheral homocysteine levels during the manic and euthymic phases [51]. The increased homocysteine levels lead to the NMDA receptor activation, causing increased intracellular calcium, ROS production, and subsequently cause neuronal autophagy or necrosis [52]. Hyperhomocysteinemia depletes the nitric oxide and antioxidants, leading to an increase in platelet-derived growth factors and the activation of clotting factors, which leads to platelet adhesion, aggregation, and thrombus formation [53]. In addition, hyperhomocysteinemia also disrupts the structural integrity of collagen, proteoglycans, and elastin, leading to arterial stiffening [54]. All these factors contribute to vessels occlusion and an increased risk of MI in patients with bipolar disorder [35].

## 4. Schizophrenia

**Figure 3:**
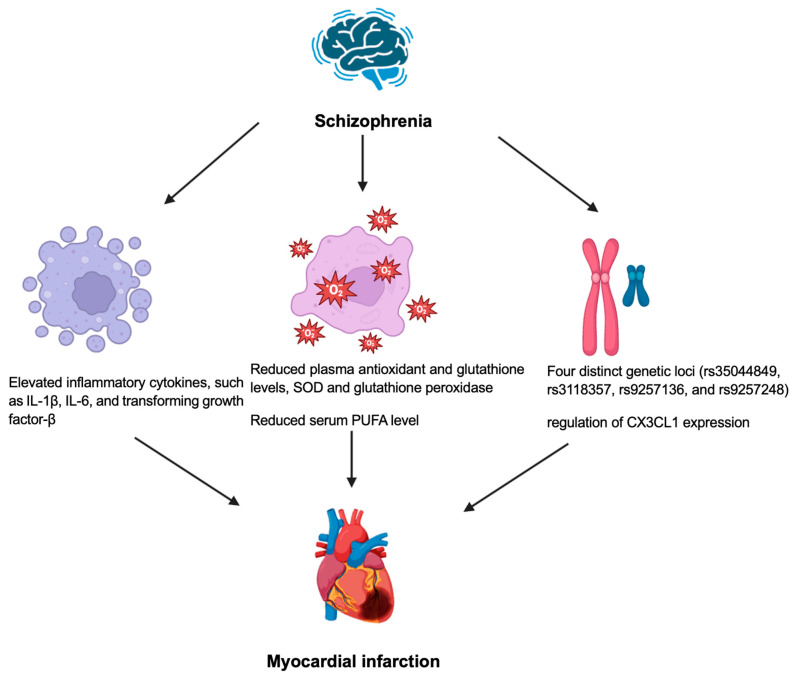
Pathophysiology of myocardial infarction and schizophrenia. IL: interleukin, PUFA: polyunsaturated fatty acid, SOD: superoxide dismutase.

Figure 3 shows the pathophysiology of myocardial infarction and schizophrenia. Inflammation and immune system dysfunction have been observed in individuals with schizophrenia [55]. Cytokines play an essential role in mediating the effects of inflammation in schizophrenia, potentially associating prenatal insults to the schizophrenia [55]. Prenatal infections and maternal immune system alterations have been identified as significant factors, increasing the likelihood of schizophrenia and related neurocognitive and neuroanatomical abnormalities in offspring [56]. Patients with psychosis may demonstrate a higher level of pro-inflammatory cytokines [57]. The activation of the inflammatory response system may lead to microglial activation, as evidenced by post-mortem studies showing an increase in the microglial density in the brains of patients with schizophrenia [58]. This process is believed to disrupt neuronal circuit development and function in the brain [59]. Cytokines may also induce neuronal apoptosis, potentially contributing to the functional brain deficits associated with schizophrenia [59]. Elevated levels of cytokines, such as IL-1β, IL-6, and the transforming growth factor-β, have been reported during acute relapses and the first episodes of schizophrenia but often normalize following antipsychotic treatment [60]. However other cytokines, including IL-12, IL-γ, TNF-α, and the soluble IL-2 receptor, remain elevated during acute relapses, first episodes, and even with antipsychotic therapy [38]. Increased levels of cytokines, including IL-1α, IL-6, IL-8, IL-12, IL-33, and IL-35, have a positive correlation with atherosclerosis and an increased risk of developing myocardial infarction [61].

Oxidative stress, characterized by an elevated reactive species and diminished antioxidant defenses, is implicated in cellular damage and has been consistently observed in schizophrenia [62]. The research assessing oxidative stress in schizophrenia has utilized several peripheral biomarkers, like reduced plasma, antioxidant and glutathione levels, and diminished antioxidant enzyme activities, including superoxide dismutase (SOD) and glutathione peroxidase [62]. Studies indicate that patients with acute coronary syndrome exhibit significantly lower SOD levels compared to healthy controls, with NSTEMI patients showing even lower levels than STEMI patients, suggesting more severe oxidative stress [63]. The reduced SOD and catalase levels may result from the increased utilization to neutralize the free radicals generated during myocardial ischemia, reflecting a depletion of antioxidant defenses [63]. In addition, approximately one-third of schizophrenia patients exhibit pronounced redox dysregulation, with acute-phase polyunsaturated fatty acid (PUFA) deficits and detrimental responses to eicosapentanoate (EPA) or vitamin E and C, although these effects stabilize during remission, marked by a persistent redox imbalance in low-PUFA subgroups [64,65]. A reduced serum PUFA level has been connected to an increased risk of cardiovascular events, including myocardial infarction [66].

Genome-wide association studies demonstrated the shared genetic variants associated with schizophrenia and cardiovascular diseases due to the similar pathophysiology of inflammation and metabolism [67]. Four distinct genetic loci (rs35044849, rs3118357, rs9257136, and rs9257248) have demonstrated significant colocalization between schizophrenia and cardiovascular disease, suggesting that these variants may influence both cardiovascular and neurological systems [68]. The shared genetic loci highlights a potential role for this locus in the heart–brain axis through the regulation of the CX3CL1 expression, which is a chemokine that is implicated in immune responses and neuroinflammation, potentially linking cardiovascular disease and schizophrenia [68]. CX3CL1 is overexpressed in atherosclerotic plaques, contributing to their formation and progression by recruiting inflammatory cells, like monocytes, to vascular walls leading to an increased risk of myocardial infarction [69]. In addition, elevated CX3CL1 levels have been observed in heart failure patients, correlating with an increased risk of cardiac dysfunction through the mechanisms involving inflammation and tissue remodeling [70]. Furthermore, the association of rs35044849 with various schizophrenia phenotypes and proHB-EGF reduces cardiac contractility, causes interstitial fibrosis and exacerbates cardiac remodeling after myocardial infarction leading to worsening cardiac function [68].

Substance use disorders, including tobacco, alcohol, cannabis, and cocaine use disorders, are frequently observed in patients with schizophrenia with a lifetime prevalence from 60% to 90% for cigarette smoking, 21% to 86% for alcohol use, and 17% to 83% for cannabis use [71]. Gene–environment interactions contribute to the risk of schizophrenia and co-occurring substance use disorders, with BDNF, catechol-O-methyltransferase (COMT), and protein kinase B (AKT) being the most studied genes linked to both conditions [71]. Furthermore, schizophrenia may stem from a dysfunction in the brain circuits related to reward and motivation, particularly the mesocorticolimbic dopamine system, leading to both substance initiation and continued use [71]. Substance use significantly contributes to an increased risk of MI, and a significant proportion of cases do not manifest any symptoms or signs of coronary artery disease, likely due to coronary microvascular dysfunction [72].

## 5. Future Research Directions

Major psychiatric disorders are linked to a reduced lifespan and signs of accelerated aging due to disruptions in circadian rhythms leading to oxidative stress, inflammation, and mitochondrial function [73]. Circadian disruptions are commonly observed in psychiatric conditions, often linked to reduced melatonin levels and the dysregulation of the HPA axis [74]. The suppression of pineal melatonin in the nighttime disrupts the regulation of the cortisol levels during sleep, affecting the morning cortisol awakening response, and the activated glucocorticoid receptor-alpha (GR-α) from moving into the nucleus [74]. The suppression of melatonin throughout the circadian cycle may disrupt the regulation of the HPA axis, affecting cardiovascular function and recovery after myocardial infarction [75]. Gut dysbiosis and the suppression of butyrate levels, which are typically evident in psychiatric conditions, reduce the butyrate’s ability to suppress the GR-α nuclear translocation from its cytoplasmic complex with Hsp-90 and p-23 [76]. Since pineal melatonin at night helps to seal the gut and prevent gut dysbiosis; therefore, its suppression may be closely linked to alterations in the gut microbiome [77]. Alterations to the gut microbiota increase the risk of myocardial infarction by modifying gut-produced metabolites and complex interplay with host genetics [78].

In addition, mood and psychotic disorders are often associated with hyperglycemia and metabolic dysregulation [79]. Hyperglycemia and hypertension increase methylglyoxal, which is typically modeled as mediating its effects through its role as a precursor for advanced glycation end-products (AGEs) and by activating the receptor for AGEs [80]. However, recent data indicate that methylglyoxal binds to and reduces the availability of tryptophan, thereby restricting the initiation of the tryptophan-serotonin-N-acetylserotonin-melatonin pathway [81]. As this pathway is also relevant to cardiovascular and cardiomyocyte function, the suppression of this pathway may, therefore, represent another overlapping pathophysiological feature linking mood/psychotic disorders with myocardial infarction [82].

## 6. Conclusions

This review highlights the bidirectional relationship between MI and major psychiatric conditions. The shared pathophysiological mechanisms, such as inflammation, oxidative stress, neurohormonal dysregulation, and genetic factors, underlie this connection between MI and psychiatric illnesses. Advancements in understanding the pathophysiological mechanisms provide critical insights regarding the complexities of managing patients with comorbid MI and psychiatric disorders. Further research is essential to identify the targeted interventions addressing these shared mechanisms to improve both mental and cardiovascular diseases. A holistic approach incorporating multidisciplinary care, early diagnosis, and innovative therapeutic strategies is essential for optimizing the outcomes in this vulnerable population.

## Data Availability

No new data were created or analyzed in this study.

## Abbreviations

The following abbreviations are used in this manuscript:

ACTH	Adrenocorticotropic hormone
AGE	Advanced glycation end products
AKT	Protein kinase B
AMPK	AMP-activated protein kinase
ANS	Autonomic nervous system
ATP	Adenosine triphosphate
BNDF	Brain-derived neurotrophic factor
COMT	catechol-O-methyltransferase
CRH	Corticotropin-releasing hormone
DAMP	Damage-associated molecular patterns
EPA	Eicosapentanoate
GR-α	Glucocorticoid receptor-alpha
HMGB1	High-mobility group box 1
HPA	Hypothalamic-pituitary-adrenal
HSP	Heat shock proteins
IL	Interleukin
LDL	Low-density lipoprotein
MI	Myocardial infarction
MTHFR	Methylenetetrahydrofolate reductase
MPTP	Mitochondrial permeability transition pore
MCU	Mitochondrial calcium uniporter
NCX	Na^+^/Ca^2^^+^ exchanger
NMDA	N-methyl-D-aspartate
NSTEMI	Non-ST elevation MI
PAI-1	Plasminogen activator inhibitor 1
PUFA	Polyunsaturated fatty acid
ROS	Reactive oxygen species
SOD	Superoxide dismutase
STEMI	ST elevation myocardial infarction
TNF	Tumor necrosis factor
tPA	tissue plasminogen activator

## References

[1] Kumar M., Nayak P.K. (2017). Psychological sequelae of myocardial infarction. Biomed. Pharmacother..

[2] Grippo A.J., Johnson A.K. (2009). Stress, depression and cardiovascular dysregulation: A review of neurobiological mechanisms and the integration of research from preclinical disease models. Stress.

[3] Ringen P.A., Engh J.A., Birkenaes A.B., Dieset I., Andreassen O.A. (2014). Increased mortality in schizophrenia due to cardiovascular disease—A non-systematic review of epidemiology, possible causes, and interventions. Front. Psychiatry.

[4] Laursen T.M., Wahlbeck K., Hällgren J., Westman J., Ösby U., Alinaghizadeh H., Gissler M., Nordentoft M. (2013). Life expectancy and death by diseases of the circulatory system in patients with bipolar disorder or schizophrenia in the Nordic countries. PLoS ONE.

[5] Lambert A.M., Parretti H.M., Pearce E., Price M.J., Riley M., Ryan R., Tyldesley-Marshall N., Avşar T.S., Matthewman G., Lee A. (2022). Temporal trends in associations between severe mental illness and risk of cardiovascular disease: A systematic review and meta-analysis. PLoS Med..

[6] Goldfarb M., De Hert M., Detraux J., Di Palo K., Munir H., Music S., Piña I., Ringen Petter A. (2022). Severe Mental Illness and Cardiovascular Disease. J. Am. Coll. Cardiol..

[7] Kugathasan P., Johansen M.B., Jensen M.B., Aagaard J., Nielsen R.E., Jensen S.E. (2019). Coronary Artery Calcification and Mortality Risk in Patients With Severe Mental Illness. Circ. Cardiovasc. Imaging.

[8] De Hert M., Detraux J., Vancampfort D. (2018). The intriguing relationship between coronary heart disease and mental disorders. Dialogues Clin. Neurosci..

[9] Feng H.P., Chien W.C., Cheng W.T., Chung C.H., Cheng S.M., Tzeng W.C. (2016). Risk of anxiety and depressive disorders in patients with myocardial infarction: A nationwide population-based cohort study. Medicine.

[10] Roest A.M., Heideveld A., Martens E.J., de Jonge P., Denollet J. (2014). Symptom dimensions of anxiety following myocardial infarction: Associations with depressive symptoms and prognosis. Health Psychol..

[11] Ricci M., Pozzi G., Caraglia N., Chieffo D.P.R., Polese D., Galiuto L. (2024). Psychological Distress Affects Performance during Exercise-Based Cardiac Rehabilitation. Life.

[12] Garrels E., Kainth T., Silva B., Yadav G., Gill G., Salehi M., Gunturu S. (2023). Pathophysiological mechanisms of post-myocardial infarction depression: A narrative review. Front. Psychiatry.

[13] Wilkowska A., Rynkiewicz A., Wdowczyk J., Landowski J. (2019). Morning and afternoon serum cortisol level in patients with post-myocardial infarction depression. Cardiol. J..

[14] Sjögren E., Leanderson P., Kristenson M. (2006). Diurnal saliva cortisol levels and relations to psychosocial factors in a population sample of middle-aged Swedish men and women. Int. J. Behav. Med..

[15] Frangogiannis N.G. (2014). The inflammatory response in myocardial injury, repair, and remodelling. Nat. Rev. Cardiol..

[16] Libby P., Ridker P.M., Hansson G.K. (2011). Progress and challenges in translating the biology of atherosclerosis. Nature.

[17] Konsman J.P., Luheshi G.N., Bluthé R.M., Dantzer R. (2000). The vagus nerve mediates behavioural depression, but not fever, in response to peripheral immune signals; a functional anatomical analysis. Eur. J. Neurosci..

[18] Penninx B.W., Kritchevsky S.B., Yaffe K., Newman A.B., Simonsick E.M., Rubin S., Ferrucci L., Harris T., Pahor M. (2003). Inflammatory markers and depressed mood in older persons: Results from the Health, Aging and Body Composition study. Biol. Psychiatry.

[19] Maes M., Bosmans E., De Jongh R., Kenis G., Vandoolaeghe E., Neels H. (1997). Increased serum IL-6 and IL-1 receptor antagonist concentrations in major depression and treatment resistant depression. Cytokine.

[20] Raison C.L., Capuron L., Miller A.H. (2006). Cytokines sing the blues: Inflammation and the pathogenesis of depression. Trends Immunol..

[21] O’Connor J.C., Lawson M.A., André C., Moreau M., Lestage J., Castanon N., Kelley K.W., Dantzer R. (2009). Lipopolysaccharide-induced depressive-like behavior is mediated by indoleamine 2,3-dioxygenase activation in mice. Mol. Psychiatry.

[22] Dantzer R., O’Connor J.C., Lawson M.A., Kelley K.W. (2011). Inflammation-associated depression: From serotonin to kynurenine. Psychoneuroendocrinology.

[23] Tsai S.J. (2017). Role of tissue-type plasminogen activator and plasminogen activator inhibitor-1 in psychological stress and depression. Oncotarget.

[24] Tsai S.J., Hong C.J., Liou Y.J., Yu Y.W., Chen T.J. (2008). Plasminogen activator inhibitor-1 gene is associated with major depression and antidepressant treatment response. Pharmacogenet Genom..

[25] Geiser F., Conrad R., Imbierowicz K., Meier C., Liedtke R., Klingmüller D., Oldenburg J., Harbrecht U. (2011). Coagulation activation and fibrinolysis impairment are reduced in patients with anxiety and depression when medicated with serotonergic antidepressants. Psychiatry Clin. Neurosci..

[26] Jiang H., Chen S., Li C., Lu N., Yue Y., Yin Y., Zhang Y., Zhi X., Zhang D., Yuan Y. (2017). The serum protein levels of the tPA-BDNF pathway are implicated in depression and antidepressant treatment. Transl. Psychiatry.

[27] Karege F., Perret G., Bondolfi G., Schwald M., Bertschy G., Aubry J.M. (2002). Decreased serum brain-derived neurotrophic factor levels in major depressed patients. Psychiatry Res..

[28] Hashimoto K. (2010). Brain-derived neurotrophic factor as a biomarker for mood disorders: An historical overview and future directions. Psychiatry Clin. Neurosci..

[29] Krishnan V., Nestler E.J. (2008). The molecular neurobiology of depression. Nature.

[30] Hofer M., Pagliusi S.R., Hohn A., Leibrock J., Barde Y.A. (1990). Regional distribution of brain-derived neurotrophic factor mRNA in the adult mouse brain. Embo J..

[31] Otte C., McCaffery J., Ali S., Whooley M.A. (2007). Association of a serotonin transporter polymorphism (5-HTTLPR) with depression, perceived stress, and norepinephrine in patients with coronary disease: The Heart and Soul Study. Am. J. Psychiatry.

[32] Schins A., Honig A., Crijns H., Baur L., Hamulyák K. (2003). Increased coronary events in depressed cardiovascular patients: 5-HT2A receptor as missing link?. Psychosom. Med..

[33] Nakatani D., Sato H., Sakata Y., Shiotani I., Kinjo K., Mizuno H., Shimizu M., Ito H., Koretsune Y., Hirayama A. (2005). Influence of serotonin transporter gene polymorphism on depressive symptoms and new cardiac events after acute myocardial infarction. Am. Heart J..

[34] Bujak M., Frangogiannis N.G. (2009). The role of IL-1 in the pathogenesis of heart disease. Arch. Immunol. Ther. Exp (Warsz).

[35] Shah D., Singh B., Varnika F., Fredrick F.C., Meda A.K.R., Aggarwal K., Jain R. (2024). Linking hearts and minds: Understanding the cardiovascular impact of bipolar disorder. Future Cardiol..

[36] La Rovere M.T., Porta A., Schwartz P.J. (2020). Autonomic Control of the Heart and Its Clinical Impact. A Personal Perspective. Front. Physiol..

[37] van Weperen V.Y.H., Ripplinger C.M., Vaseghi M. (2023). Autonomic control of ventricular function in health and disease: Current state of the art. Clin. Auton. Res..

[38] Goldstein B.I., Carnethon M.R., Matthews K.A., McIntyre R.S., Miller G.E., Raghuveer G., Stoney C.M., Wasiak H., McCrindle B.W. (2015). Major Depressive Disorder and Bipolar Disorder Predispose Youth to Accelerated Atherosclerosis and Early Cardiovascular Disease: A Scientific Statement From the American Heart Association. Circulation.

[39] Henry B.L., Minassian A., Paulus M.P., Geyer M.A., Perry W. (2010). Heart rate variability in bipolar mania and schizophrenia. J. Psychiatr. Res..

[40] Hillebrand S., Gast K.B., de Mutsert R., Swenne C.A., Jukema J.W., Middeldorp S., Rosendaal F.R., Dekkers O.M. (2013). Heart rate variability and first cardiovascular event in populations without known cardiovascular disease: Meta-analysis and dose–response meta-regression. EP Eur..

[41] Fatkhullina A.R., Peshkova I.O., Koltsova E.K. (2016). The Role of Cytokines in the Development of Atherosclerosis. Biochem. (Mosc).

[42] Steckert A.V., Valvassori S.S., Moretti M., Dal-Pizzol F., Quevedo J. (2010). Role of oxidative stress in the pathophysiology of bipolar disorder. Neurochem. Res..

[43] Goldstein B.I. (2017). Bipolar Disorder and the Vascular System: Mechanisms and New Prevention Opportunities. Can. J. Cardiol..

[44] Juan C.A., Pérez de la Lastra J.M., Plou F.J., Pérez-Lebeña E. (2021). The Chemistry of Reactive Oxygen Species (ROS) Revisited: Outlining Their Role in Biological Macromolecules (DNA, Lipids and Proteins) and Induced Pathologies. Int. J. Mol. Sci..

[45] Checa J., Aran J.M. (2020). Reactive Oxygen Species: Drivers of Physiological and Pathological Processes. J. Inflamm. Res..

[46] Wu X., Li J., Cheng H., Wang L. (2024). Ferroptosis and Lipid Metabolism in Acute Myocardial Infarction. Rev. Cardiovasc. Med..

[47] Morris G., Walder K., McGee S.L., Dean O.M., Tye S.J., Maes M., Berk M. (2017). A model of the mitochondrial basis of bipolar disorder. Neurosci. Biobehav. Rev..

[48] Cantó C., Menzies K.J., Auwerx J. (2015). NAD(+) Metabolism and the Control of Energy Homeostasis: A Balancing Act between Mitochondria and the Nucleus. Cell Metab..

[49] Hernandez-Resendiz S., Prunier F., Girao H., Dorn G., Hausenloy D.J. (2020). Targeting mitochondrial fusion and fission proteins for cardioprotection. J. Cell Mol. Med..

[50] De Stefani D., Raffaello A., Teardo E., Szabò I., Rizzuto R. (2011). A forty-kilodalton protein of the inner membrane is the mitochondrial calcium uniporter. Nature.

[51] Salagre E., Vizuete A.F., Leite M., Brownstein D.J., McGuinness A., Jacka F., Dodd S., Stubbs B., Köhler C.A., Vieta E. (2017). Homocysteine as a peripheral biomarker in bipolar disorder: A meta-analysis. Eur. Psychiatry.

[52] Ganguly P., Alam S.F. (2015). Role of homocysteine in the development of cardiovascular disease. Nutr. J..

[53] Yuan S., Mason A.M., Carter P., Burgess S., Larsson S.C. (2021). Homocysteine, B vitamins, and cardiovascular disease: A Mendelian randomization study. BMC Med..

[54] Namazi M.R., Feily A. (2011). Homocysteine may accelerate skin aging: A new chapter in the biology of skin senescence?. J. Am. Acad. Dermatol..

[55] Henderson D.C., Vincenzi B., Andrea N.V., Ulloa M., Copeland P.M. (2015). Pathophysiological mechanisms of increased cardiometabolic risk in people with schizophrenia and other severe mental illnesses. Lancet Psychiatry.

[56] Cheslack-Postava K., Brown A.S. (2022). Prenatal infection and schizophrenia: A decade of further progress. Schizophr. Res..

[57] Fond G., Lançon C., Korchia T., Auquier P., Boyer L. (2020). The Role of Inflammation in the Treatment of Schizophrenia. Front. Psychiatry.

[58] van Kesteren C.F., Gremmels H., de Witte L.D., Hol E.M., Van Gool A.R., Falkai P.G., Kahn R.S., Sommer I.E. (2017). Immune involvement in the pathogenesis of schizophrenia: A meta-analysis on postmortem brain studies. Transl. Psychiatry.

[59] Monji A., Kato T., Kanba S. (2009). Cytokines and schizophrenia: Microglia hypothesis of schizophrenia. Psychiatry Clin. Neurosci..

[60] Miller B.J., Buckley P., Seabolt W., Mellor A., Kirkpatrick B. (2011). Meta-analysis of cytokine alterations in schizophrenia: Clinical status and antipsychotic effects. Biol. Psychiatry.

[61] Haybar H., Bandar B., Torfi E., Mohebbi A., Saki N. (2023). Cytokines and their role in cardiovascular diseases. Cytokine.

[62] Murray A.J., Rogers J.C., Katshu M., Liddle P.F., Upthegrove R. (2021). Oxidative Stress and the Pathophysiology and Symptom Profile of Schizophrenia Spectrum Disorders. Front. Psychiatry.

[63] Aladağ N., Asoğlu R., Ozdemir M., Asoğlu E., Derin A.R., Demir C., Demir H. (2021). Oxidants and antioxidants in myocardial infarction (MI): Investigation of ischemia modified albumin, malondialdehyde, superoxide dismutase and catalase in individuals diagnosed with ST elevated myocardial infarction (STEMI) and non-STEMI (NSTEMI). J. Med. Biochem..

[64] Solberg D.K., Refsum H., Andreassen O.A., Bentsen H. (2019). A five-year follow-up study of antioxidants, oxidative stress and polyunsaturated fatty acids in schizophrenia. Acta Neuropsychiatr..

[65] Bentsen H., Landrø N.I. (2018). Neurocognitive effects of an omega-3 fatty acid and vitamins E+C in schizophrenia: A randomised controlled trial. Prostaglandins Leukot. Essent. Fat. Acids.

[66] Yagi S., Fukuda D., Aihara K.I., Akaike M., Shimabukuro M., Sata M. (2017). n-3 Polyunsaturated Fatty Acids: Promising Nutrients for Preventing Cardiovascular Disease. J. Atheroscler. Thromb..

[67] Lam M., Chen C.Y., Li Z., Martin A.R., Bryois J., Ma X., Gaspar H., Ikeda M., Benyamin B., Brown B.C. (2019). Comparative genetic architectures of schizophrenia in East Asian and European populations. Nat. Genet..

[68] Shen J., Jiang C. (2024). Unraveling the heart-brain axis: Shared genetic mechanisms in cardiovascular diseases and Schizophrenia. Schizophrenia.

[69] Apostolakis S., Spandidos D. (2013). Chemokines and atherosclerosis: Focus on the CX3CL1/CX3CR1 pathway. Acta Pharmacol. Sin..

[70] Liu W., Jiang L., Bian C., Liang Y., Xing R., Yishakea M., Dong J. (2016). Role of CX3CL1 in Diseases. Arch. Immunol. Ther. Exp (Warsz).

[71] Khokhar J.Y., Dwiel L.L., Henricks A.M., Doucette W.T., Green A.I. (2018). The link between schizophrenia and substance use disorder: A unifying hypothesis. Schizophr. Res..

[72] Jalali Z., Khademalhosseini M., Soltani N., Esmaeili Nadimi A. (2021). Smoking, alcohol and opioids effect on coronary microcirculation: An update overview. BMC Cardiovasc. Disord..

[73] Anderson G. (2023). Melatonin, BAG-1 and cortisol circadian interactions in tumor pathogenesis and patterned immune responses. Explor. Target. Antitumor Ther..

[74] Serrano-Serrano A.B., Marquez-Arrico J.E., Navarro J.F., Martinez-Nicolas A., Adan A. (2021). Circadian Characteristics in Patients under Treatment for Substance Use Disorders and Severe Mental Illness (Schizophrenia, Major Depression and Bipolar Disorder). J. Clin. Med..

[75] Sethi Y., Padda I., Sebastian S.A., Malhi A., Malhi G., Fulton M., Khehra N., Mahtani A., Parmar M., Johal G. (2023). Glucocorticoid Receptor Antagonism and Cardiomyocyte Regeneration Following Myocardial Infarction: A Systematic Review. Curr. Probl. Cardiol..

[76] Hadrich I., Turki M., Chaari I., Abdelmoula B., Gargouri R., Khemakhem N., Elatoui D., Abid F., Kammoun S., Rekik M. (2025). Gut mycobiome and neuropsychiatric disorders: Insights and therapeutic potential. Front. Cell. Neurosci..

[77] Zhang B., Chen T., Cao M., Yuan C., Reiter R.J., Zhao Z., Zhao Y., Chen L., Fan W., Wang X. (2022). Gut Microbiota Dysbiosis Induced by Decreasing Endogenous Melatonin Mediates the Pathogenesis of Alzheimer’s Disease and Obesity. Front. Immunol..

[78] Bijla M., Saini S.K., Pathak A.K., Bharadwaj K.P., Sukhavasi K., Patil A., Saini D., Yadav R., Singh S., Leeuwenburgh C. (2024). Microbiome interactions with different risk factors in development of myocardial infarction. Exp. Gerontol..

[79] Penninx B., Lange S.M.M. (2018). Metabolic syndrome in psychiatric patients: Overview, mechanisms, and implications. Dialogues Clin. Neurosci..

[80] Vangrieken P., Scheijen J., Schiffers P.M.H., van de Waarenburg M.P.H., Foulquier S., Schalkwijk C.C.G. (2025). Modelling the effects of elevated methylglyoxal levels on vascular and metabolic complications. Sci. Rep..

[81] Md S., Hong S.M., Lee J.H., Park H., Chang K.A., Kim H.B., Park M.G., Eo H., Oh M.S., Kim S.Y. (2024). Depression like-behavior and memory loss induced by methylglyoxal is associated with tryptophan depletion and oxidative stress: A new in vivo model of neurodegeneration. Biol. Res..

[82] Manjarrez-Gutiérrez G., Valero-Elizondo G., Serrano-Hernández Y., Mondragón-Herrera J.A., Mansilla-Olivares A. (2022). Hypertrophic cardiomyopathy induces changes in the tryptophan-5-hydroxylase, serotonin transporter and serotonergic receptors expressions. Gac. Med. Mex..

